# Metastasis of cN0 Papillary Thyroid Carcinoma of the Isthmus to the Lymph Node Posterior to the Right Recurrent Laryngeal Nerve

**DOI:** 10.3389/fendo.2021.677986

**Published:** 2021-05-10

**Authors:** Wei Du, Qigen Fang, Xu Zhang, Liyuan Dai

**Affiliations:** Department of Head Neck and Thyroid, Affiliated Cancer Hospital of Zhengzhou University, Henan Cancer Hospital, Zhengzhou, China

**Keywords:** papillary thyroid carcinoma, thyroid isthmus, lymph node posterior to the right recurrent laryngeal nerve, central lymph node metastasis, lymph node anterior to the right recurrent laryngeal nerve

## Abstract

**Objective:**

The association between metastasis to the lymph node posterior to the right recurrent laryngeal nerve (LN-prRLN) and cN0 papillary thyroid carcinoma (PTC) located in the thyroid isthmus remains unknown; therefore, our goal was to analyze the characteristics of LN-prRLN metastasis of cN0 PTCs of the thyroid isthmus and determine its potential predictors.

**Patients and methods:**

This retrospective study included patients who underwent bilateral central neck dissection between January 2018 and January 2021. The specimen was divided into five groups of prelaryngeal lymph node (LN), pretracheal LN, left paratracheal LN, lymph node anterior to the right recurrent laryngeal nerve (LN-arRLN), and LN-prRLN. Univariate and multivariate analyses were used to assess the association between the clinical pathologic variables and LN-prRLN metastases. Surgical complications were presented descriptively.

**Results:**

A total of 357 patients were included, LN-prRLN metastasis occurred in 23 (6.4%) patients, and LN-prRLN was positive only when there were other LN metastases, especially LN-arRLN metastases. Other independent risk factors for LN-prRLN included foci numbers ≥2, tumor size ≥5.0 mm, and extrathyroidal extensions. The rates of permanent hypoparathyroidism and vocal cord paralysis were 1.1% and 2.0%, respectively.

**Conclusion:**

LN-prRLN metastases should not be ignored in cN0 PTC located in the thyroid isthmus; however, its dissection is a safe procedure, and the status of LN-arRLN can be a reliable predictor for LN-prRLN metastases.

## Introduction

Of all thyroid cancers, papillary thyroid carcinoma (PTC) is the most common histologic type. It has a relatively high occult central neck lymph node (LN) metastasis rate ranging from 25.7% to 60% (1–7). The importance of prophylactic central neck dissections (CNDs) in PTCs has been confirmed by a recent ATA guideline (8). Central neck LNs consist of prelaryngeal (Delphian) LNs, pretracheal LNs, and paratracheal LNs. Based on anatomical differences, the right paratracheal LNs are divided into two subgroups: LN posterior to the right recurrent laryngeal nerve (LN-prRLN) and LN anterior to the right recurrent laryngeal nerve (LN-arRLN). Dissection of the LN-prRLN requires patience and experience and could lead to nerve damage, affecting the patient’s quality of life. Its use has been controversial and often overlooked.

However, there is increasing evidence that LN-prRLN metastases can occur in up to 26.6% of cN0 PTCs and that an additional removal procedure is not associated with an increased possibility of transient or permanent hypoparathyroidism or vocal cord paralysis (9–15). Common risk factors for LN-prRLN metastases include multiple foci, extrathyroidal extensions (ETE), right lobe tumors, tumor size, LN-arRLN metastases, and the number of positive LNs in the central neck. However, these finding were based on studies that included only patients with PTCs located in the right lobe (9) or with PTC, irrespective of tumor site (7, 10–12). Studies on patients with PTCs located in the isthmus had very limited sample sizes (13–15). However, PTCs located in the thyroid isthmus have significantly different pathologic features. They present with a higher incidence of multifocality, invasions of the thyroid capsules and adjacent tissues, and central node involvement compared to carcinomas located in other parts of the thyroid (16). Therefore, in current study, we aimed to analyze the metastatic characteristics of the LN-prRLN and its potential predictors in PTC located in the thyroid isthmus.

## Patients and Methods

### Ethical *C*onsiderations

Our hospital Institutional Research Committee approved this study, and all the participants signed an informed consent agreement. All the procedures involving human participants were conducted in accordance with the ethical standards of the Institutional and/or National Research Committee and the 1964 Helsinki Declaration and its later amendments or comparable ethical standards.

### Patient Selection

Medical records of all the patients (>18 years) who received surgical treatment for PTC between January 2018 and January 2021 were reviewed retrospectively. The selection criteria were as follows: having a primary PTC confirmed by postoperative pathology; location of the PTC in the thyroid isthmus without any suspicious malignant nodules in the bilateral thyroid lobes, confirmed with preoperative ultrasonography; presence of cN0 neck, confirmed with preoperative ultrasonography and computed tomography (CT). Patients with previous neck surgeries, family histories of cancer, and histories of neck radiotherapy were excluded. The following data were extracted and analyzed for the included patients: demographic data; pathologies including ETE, capsular invasions, perineural invasions (PNIs), and lymphovascular invasions (LVIs); BRAF 600E mutations; and postoperative complications.

### Definitions of Important Variables

An isthmus PTC was defined as a tumor with its boundaries within the lateral borders of the trachea (17). A cN0 neck was defined as a neck which did not have following features on ultrasonography: the absence of an echogenic hilum, a round shape, microcalcification, peripheral blood flow on color Doppler images, and cystic changes (18) and on CT: areas with clear evidence of nonfat, low-density, or liquid components; the largest diameter >15 mm at level II and >10 mm at other levels; and ratios of the longest to smallest diameter ≤2 (19). Tumor size was defined as the longest diameter of the tumor.

Capsular invasion was defined as the invasion of the thyroid capsular by a tumor and ETE as tumor invasion of structures outside the thyroid, including the trachea, anterior cervical muscle, larynx, and cricothyroid muscle. Both capsular invasion and ETE were evaluated based on intraoperative findings and postoperative pathologies.

### Surgical Treatment Procedures

There was no specific guideline for treating a cN0 PTC located in the thyroid isthmus in China. In our cancer center, there are two procedures available, total thyroidectomy and wide field isthmusectomy. The choice of treatment method depended on the surgeon’s preference and the patient’s condition.

From January 2018, a prophylactic bilateral CND was performed for all patients with cN0 PTCs located in the thyroid isthmus. The surgical specimens of patients were divided into five subgroups: prelaryngeal LN, pretracheal LN, left paratracheal LN, LN-arRLN, and LN-prRLN. A pathologic analysis was conducted separately for every patient.

### Statistical Analysis

The association between the clinical pathologic variables and LN-prRLN metastases was assessed first using a Chi-square test, and then to find the independent predictors, a multivariate analysis of the variables that were found to be significant in the univariate analysis was performed. All the statistical analyses were performed using SPSS 20.0, and a *P*<0.05 was considered to be significant.

## Results

### Baseline Data of the Enrolled Patients

A total of 357 patients were included in the study; of these patients, 281 (78.7%) were female and 76 (21.3%) male. The mean age was 47.6 years with a range of 20 to 68 years. The mean tumor size was 7.7 mm with a range from 1.7 to 13.6 mm. Coexistent Hashimoto’s disease was found in 103 (28.9%) patients and BRAF 600E mutations were noted in 257 (72.0%) patients. Multiple foci (≥2) occurred in 31 (8.7%) patients, while ETE and capsular invasions were noted in 48 (13.4%) and 78 (21.8%) patients, respectively. PNI and LVI were observed in 26 (7.3%) and 21 (5.9%) patients, respectively. Total thyroidectomy and wide field isthmusectomy were performed in 204 (57.1%) and 153 (42.9%) patients, respectively.

### Central LN Metastatic Characteristics

Central LN metastases were noted in 129 (36.1%) patients. Prelaryngeal and pretracheal LN metastases occurred in 60 (16.8%) and 56 (15.7%) patients, respectively. Forty six (12.9%) patients had left paratracheal LN metastases, while LN-arRLN and LN-prRLN metastases occurred in 52 (14.6%) and 23 (6.4%) patients, respectively.

**Table 1** describes the metastatic pattern in the 129 patients. Prelaryngeal LN metastasis alone was the most common pattern, which occurred in 38.8% of the cases, followed by a pattern of concurrent metastases of the pretracheal LN, left paratracheal LN, and LN-arRLN. The two least common patterns were the metastases of the LN-arRLN and LN-prRLN and the metastases of the pretracheal LN and left paratracheal LN. There was no case of LN-prRLN metastasis alone.

**Table 1:** Pattern of the central lymph node (LN) metastasis in the 129 patients.

Metastatic pattern	Number of patients (%)
Prelaryngeal LN	50 (38.8%)
Pretracheal LN, left Paratracheal LN, LN-arRLN*	24 (18.6%)
Left Paratracheal LN, LN-arRLN, LN-prRLN^#^	21 (16.3%)
Pretracheal LN	16 (12.4%)
Prelaryngeal LN, Pretracheal LN	10 (7.8%)
Pretracheal LN, LN-arRLN	5 (3.9%)
LN-arRLN, LN-prRLN	2 (1.6%)
Pretracheal LN, left Paratracheal LN	1 (0.8%)

There were no overlapping numbers among each pattern.

*LN-arRLN, lymph node anterior to the right recurrent laryngeal nerve;

^#^LN-prRLN, lymph node posterior to the right recurrent laryngeal nerve.

### Predictor for LN-prRLN Metastases

The results of the univariate analysis (**Table 2**) showed that male patients had a LN-prRLN metastasis rate of 11.8%, which was significantly higher than the 5.0% observed in female patients (*P* = 0.038). In the 31 patients with multiple foci, 19.4% had LN-prRLN metastases, which was statistically different from the rate observed in 326 patients with solitary foci (*P* = 0.009). Fourteen (9.9%) of the 141 patients with tumor size ≥ 5.0 mm and 4.2% of the 216 patients with tumor size <5.0 mm had LN-prRLN metastases. The difference was significant (*P* = 0.030). A positive LN-prRLN was observed in 18.8% of patients with ETE. The rate was significantly higher than the 4.5% seen in patients without ETE (*P*=0.001). Of the patients with LN-arRLN metastases, 44.2% also had LN-prRLN metastases, and none of the patients without LN-arRLN metastases showed LN-prRLN metastases. The difference was significant (*P*<0.001).

**Table 2:** Univariate analysis of predictor for lymph node posterior to the right recurrent nerve metastasis (LN-prRLN).

Variables	LN-prRLN metastasis		p
	Positive (n = 23)	Negative (n = 334)	
Age			
<55	10 (5.3%)	180 (94.7%)	
≥55	13 (7.8%)	154 (92.2%)	0.333
Sex			
Female	14 (5.0%)	267 (95.0%)	
Male	9 (11.8%)	67 (88.2%)	0.038
Foci number			
1	17 (5.2%)	309 (94.8%)	
≥2	6 (19.4%)	25 (80.6%)	0.009
Hashimoto’s disease			
No	15 (5.9%)	239 (94.1%)	
Yes	8 (7.8%)	95 (92.2%)	0.516
Tumor size			
<5.0 mm	9 (4.2%)	207 (95.8%)	
≥5.0 mm	14 (9.9%)	127 (90.1%)	0.030
BRAF 600E mutation			
No	6 (6.0%)	94 (94.0%)	
Yes	17 (6.6%)	240 (93.4%)	0.832
ETE*			
No	14 (4.5%)	295 (95.5%)	
Yes	9 (18.8%)	39 (81.2%)	0.001
Capsular invasion			
No	19 (6.8%)	260 (93.2%)	
Yes	4 (5.1%)	74 (94.9%)	0.624
PNI^#^			
No	21 (6.3%)	310 (93.7%)	
Yes	2 (7.7%)	24 (92.3%)	1.000
LVI^!^			
No	21 (6.3%)	315 (93.7%)	
Yes	2 (9.5%)	19 (90.5%)	0.636
Prelaryngeal LN^&^ metastasis			
No	23 (7.7%)	274 (92.3%)	
Yes	0	60 (100%)	0.037
Pretracheal LN metastasis			
No	22 (7.3%)	279 (92.7%)	
Yes	1 (1.8%)	55 (98.2%)	0.148
Left paratracheal LN metastasis			
No	2 (0.6%)	309 (99.4%)	
Yes	21 (45.7%)	25 (54.3%)	<0.001
LN-arRLN^ metastasis			
No	0	305 (100%)	
Yes	23 (44.2%)	29 (55.8%)	<0.001

*ETE, extrathyroidal extension; ^#^PNI, perineural invasion; ^!^LVI, lymphovascular invasion; ^&^LN, lymph node; ^ LN-arRLN, lymph node anterior to the right recurrent laryngeal nerve.

The multivariate analysis showed that foci number ≥2, tumor size ≥5.0 mm, ETE, and LN-arRLN metastases were associated with an increased possibility of LN-prRLN metastases (all *P*<0.05, **Table 3**).

**Table 3:** Multivariate analysis predictor for lymph node posterior to the right recurrent nerve metastasis.

Variables	p	OR [95% CI]
Sex	0.134	3.674 [0.663–9.552]
Foci number	0.021	2.185 [1.291–8.335]
Tumor size	0.004	2.675 [1.472–9.423]
ETE*	<0.001	5.765 [1.892–16.448]
Prelaryngeal LN^&^ metastasis	0.455	0.689 [0.112–10.382]
left Paratracheal LN metastasis	0.217	3.288 [0.785–11.332]
LN-arRLN^ metastasis	<0.001	6.876 [2.118–20.674]

*ETE, extrathyroidal extension; ^&^LN, lymph node; ^LN-arRLN, lymph node anterior to the right recurrent laryngeal nerve.

### Postoperative Complication

Transient hypoparathyroidism occurred in 47 patients (13.2%), and 43 cases gradually returned to be normal within 6 months after surgery, while four patients had permanent hypoparathyroidism, which was equivalent to a rate of 1.1%.

Hoarseness occurred in 17 (4.8%) patients immediately after surgery. During follow-up, the voice of 10 patients recovered without vocal cord immobility confirmed by a direct laryngoscope; four patients had a normal voice with vocal cord paralysis, while three patients showed consistent voice change with vocal cord immobility.

## Discussion

To our best knowledge, this was the first study to analyze the features of LN-prRLN metastasis of cN0 PTCs located in the thyroid isthmus. A metastasis rate of 6.4% was observed, and there was no skip metastasis of LN-prRLN. The status of LN-arRLN can be used as a reliable predictor for LN-prRLN metastases, and the dissection of the LN-prRLN did not increase the possibility of recurrent laryngeal nerve and parathyroid gland damage occurring.

A study by Grodski et al. found that LN-prRLN resection may be recommended in routine CNDs (20); however, this procedure was related to an increased risk of nerve injury due to the traction and elevation that occurs during the LN removal. Therefore, a series of researchers explored the incidence of LN-prRLN metastases, and the safety and necessity of LN-prRLN dissections. Lee et al. (13) enrolled 123 PTC patients, including 86 cases staged as T2-T4, and reported that 14 patients showed metastases in the LN-prRLN with an overall rate of 11.4%. Furthermore, all these patients had LN-arRLN metastases while skip metastases were not found. In addition, the T2-T4 tumors had a metastasis rate of 13.9%, which was comparable to the 5.4% found in T1 tumors. In a study by Liu et al. (15), which had a comparable sample size to that of Lee et al.’s study, the LN-prRLN metastasis rate was 11.0% in 145 PTC patients; however, four patients were negative for LN-arRLN metastasis.

However, several studies have reported higher rates and different findings than those of ours. Luo et al. (12) performed a prospective study that included 595 PTC patients, of whom a total of 102 (17.1%) patients had LN-prRLN metastases, and of these patients, 52 showed concurrent LN-arRLN negativity. In a large-scale study by Pinyi et al. (1) comprising 405 patients, LN-prRLN positivity was observed in 108 patients at a rate of 26.7%. The authors also noted that 26 patients showed LN-prRLN metastases without LN-arRLN positivity. Another large sample size study by Li et al. (10) found that LN-prRLN metastases occurred in 124 (15.1%) PTC patients, and of the 434 patients without LN-arRLN metastases, 41 (9.4%) presented LN-prRLN positivity. In a study by Chang et al. (21), which had the largest cohort size till date, of the 5556 enrolled patients, 148 were positive for LN-prRLN metastases and six did not have LN-arRLN metastases. The findings of these studies suggested that over 10% of PTC patients could have had a residual LN-prRLN disease if the dissections were not performed; however, we discovered that all of the authors had considered cN0, cN1a, and cN1b patients together. Thus, in theory, cN1 patients had increased possibility of occurrence of LN-prRLN metastases as well as that of skip metastases, making it acceptable that our incidence of LN-prRLN metastasis was 6.4% and that there was no skip metastasis observed.

A few authors have also evaluated the LN-prRLN metastases in cN0 patients. Li et al.(7) and Ito et al. (9) found that 28 (8.3%) of 338 patients and 127 (14%) of 922 patients had LN-prRLN metastases, respectively. These rates were still a little higher than the rates of our study. Usually PTCs located in the thyroid isthmus have more aggressive biologic behavior than that of PTCs arising from other sites of the thyroid (16). This viewpoint was supported by the fact that our overall central LN metastasis rate was 36.1%, which was higher than the rates in the studies by Li et al. (7) and Ito et al. (9). Moreover, the two most common metastatic sites were prelaryngeal and pretracheal LNs in the current study, which was consistent with results of previous similar studies (22). Our lower LN-prRLN metastasis rate may be explained by the small tumor size and lower multiplicity.

The safety of LN-prRLN dissection is a point of concern. Wang et al. (11) divided 1487 patients into two groups: the 378 patients who underwent CND with LN-prRLN dissection were defined as group A and the 1109 patients who underwent CND without LN-prRLN dissection as group B. Although group A had a higher incidence of hypocalcemia (7.4% *vs.* 4.0%, *P*=0.012), the two groups had similar rates of persistent hypocalcemia, transient and permanent recurrent laryngeal nerve palsies, chyle leakages, hematomas, and wound infections. Pinyi et al. (1) reported that transient recurrent laryngeal nerve injuries and hypoparathyroidism occurred in 1.0% and 1.7% patients in their cohort, respectively, and there was no persistent dysfunction. Similar results were also reported by Lee et al. (13), Yu et al. (14), Chang et al. (21), and us. All of these finding suggested LN-prRLN dissection is safe if it is performed patiently and meticulously. However, we must keep in mind that the daily life of the patients might be significantly affected if there was permanent dysfunction (23), some improvements had been introduced. Calò et al. (24) had commented intraoperative neuromonitoring had a very high sensitivity and negative predictive value, but also good specificity and positive predictive value in predicting postoperative nerve function during thyroid surgery and could assist the surgeon in intraoperative decision making. Docimo et al. (25) introduced a good option of total thyroidectomy without prophylactic central neck dissection combined with routine oral calcium and vitamin D supplements in selected differentiated thyroid cancer patients to prevent postoperative hypocalcemia for increasing the likelihood of a safe and early discharge from the hospital.

Risk factors for LN-prRLN metastases have been analyzed frequently. Age < 45 years, male sex, tumor sizes>1 cm, lateral LN metastases, ETE, multifocalities, capsule invasions, LN-arRLN metastases, and central LN metastases were all reported to be associated with LN-prRLN metastases (22). Our findings also supported these conclusions. Moreover it was noted that LN-prRLN was positive only when there were other LNs metastases, especially LN-arRLN metastasis. This finding is highly significant as it indicates that an intraoperative pathologic examination of the LN-arRLN could determine which patients would require LN-prRLN dissection.

This study had some limitations. First, this was a retrospective study with an inherent selection bias. Second, our sample size was relatively small, and more studies are required to validate the findings. Third, without a control group or survival data, our statistical power was decreased.

In summary, while LN-prRLN metastases of cN0 PTC located in the thyroid isthmus is not common, it should not be ignored; its dissection was found to be a safe procedure. LN-arRLN metastases were the most significant risk factor for LN-prRLN metastases, and it could act as a reliable predictor for LN-prRLN metastases.

## Data Availability Statement

The original contributions presented in the study are included in the article/supplementary material. Further inquiries can be directed to the corresponding author.

## Ethics Statement

The studies involving human participants were reviewed and approved by Henan Cancer Hospital ethics committee. The patients/participants provided their written informed consent to participate in this study.

## Author Contributions

All the authors made the contribution in study design, manuscript writing, studies selecting, data analysis, study quality evaluating, and manuscript revising. All authors contributed to the article and approved the submitted version.

## Conflict of Interest

The authors declare that the research was conducted in the absence of any commercial or financial relationships that could be construed as a potential conflict of interest.
